# Economic burden of diagnosed pertussis among individuals with asthma or chronic obstructive pulmonary disease in the USA: an analysis of administrative claims

**DOI:** 10.1017/S0950268817000887

**Published:** 2017-05-02

**Authors:** P. O. BUCK, J. L. MEYERS, L.-D. GORDON, R. PARIKH, S. K. KUROSKY, K. L. DAVIS

**Affiliations:** 1 GSK, 5 Crescent Drive, Philadelphia, PA, 19112, USA; 2 RTI Health Solutions, Research Triangle Park, 3040 Cornwallis Road, Post Office Box 12194, NC, 27709, USA

**Keywords:** Asthma, chronic obstructive pulmonary disease, economic burden, pertussis, retrospective database analysis

## Abstract

Individuals with chronic respiratory conditions may be at increased risk for pertussis. We conducted a retrospective administrative claims analysis to examine the incidence and economic burden of diagnosed pertussis among adolescents and adults in the USA with chronic obstructive pulmonary disease (COPD) or asthma. Patients aged ⩾11 years with diagnosed pertussis and pre-existing COPD (*n* = 343) or asthma (*n* = 1041) were matched 1:1 to patients with diagnosed pertussis but without COPD or asthma. Differences in all-cause costs (‘excess’ costs) during the 45-day and 3-month and 6-month periods before and after the pertussis index date were calculated; adjusted excess costs were estimated via multivariate regressions. The incidence of diagnosed pertussis was higher among patients with COPD or asthma than among matched patients. Compared with matched patients, patients with pertussis and pre-existing COPD or asthma accrued greater all-cause adjusted costs across study periods ($3694 and $1193 more, respectively, in the 45-day period; $4173 and $1301 more in the 3-month period; and $6154 and $1639 more in the 6-month period; all *P* < 0·0001). Patients with pre-existing COPD or asthma experience an increased economic burden after diagnosed pertussis and may especially benefit from targeted tetanus, diphtheria, and acellular pertussis vaccination strategies.

## INTRODUCTION

Pertussis is a highly communicable respiratory illness caused by the *Bordetella pertussis* bacterium, characterized by a prolonged paroxysmal cough and inspiratory whoop. After the introduction of the pertussis vaccine, the incidence of pertussis in the USA dropped substantially (from 150 cases per 100 000 persons between 1940 and 1945 to a low of approximately 1 per 100 000 persons in 1980–1990) [1]. However, recent surveillance statistics indicate a resurgence of pertussis in communities nationwide. In 2012, more than 48 000 cases of pertussis (incidence: 15·4 per 100 000 persons) were reported to the Centers for Disease Control and Prevention (CDC), an increase from 2011 (incidence: 6·1 per 100 000 population) [2]. Infants younger than 12 months of age had the highest incidence (126·7 cases per 100 000 persons), followed by adolescents aged 11–14 years (59·2 per 100 000). Despite the high incidence of pertussis in infants and children, more than half of all reported cases in 2012 occurred in persons aged 11 years and older [3].

Research has shown that the clinical presentation of pertussis in adolescents and adults is characterized by symptoms including paroxysm, whoop, posttussive apnea, and posttussive vomiting [4]. Among adolescents and adults, complications such as pneumonia, fainting, urinary incontinence, and hospitalization have been shown to increase with age [5]. Furthermore, costs associated with pertussis in adults are substantial, with previous studies estimating costs per pertussis episode among patients aged 50 years and older in the USA to be between $1800 and $14 500, depending on the treatment setting [6].

As pertussis is an infection of the respiratory tract, patients with chronic respiratory conditions such as asthma or chronic obstructive pulmonary disease (COPD) may be at increased risk for pertussis. There is some evidence showing that patients with asthma have a higher incidence of pertussis than patients without asthma [5, 7, 8]. In addition, a small case–control study (*N* = 90) found that anti-pertussis toxin immunoglobulin G seropositivity was significantly higher in patients with COPD than in control patients [9]; however, the burden of pertussis in patients with COPD requires further characterization. To bridge this research gap and provide important information on the burden of pertussis in potential high-risk groups, the objective of this study was to generate US-specific data on the incidence and economic burden of diagnosed pertussis among adolescents and adults with a pre-existing diagnosis of asthma or COPD.

## METHODS

### Study design and data source

A retrospective cohort study using US administrative claims (i.e., inpatient, outpatient, and pharmacy claims) was conducted. The data sources were the MarketScan Commercial Claims and Encounters and Medicare Supplemental and Coordination of Benefits (1/2006–6/2014) and Medicaid Multi-State (1/2007–12/2013) databases. The authors’ data access was restricted to the HIPAA (Health Insurance Portability and Accountability Act)-compliant de-identified data.

### Patient selection criteria and study cohorts

Patients were selected for the study cohorts based on standard claims-based algorithms. Specifically, patients were identified for inclusion in the analysis if they had a primary or secondary diagnosis of pertussis (defined by International Classification of Diseases, Ninth Revision, Clinical Modification (ICD-9-CM) codes 033·0x, 033·9x, and 484·3x [6]). To allow for inclusion of health care resources related to the workup of the pertussis diagnosis (e.g., initial office visits and laboratory tests), the index date was defined as the date of the first observed pertussis diagnosis minus 15 days. Patients were required to have at least 6 months of continuous enrollment before and after the index date and to be aged 11 years or older.

The pre-existing COPD and pertussis (COPD + PERT) cohort included patients with at least two claims (i.e., inpatient, emergency department, or outpatient) spaced at least 14 days apart with a COPD diagnosis (ICD-9-CM codes 491.xx, 492.xx, or 496.xx) and at least one COPD diagnosis in the 6 months before the index date. A previous study required multiple COPD claims to define exposure status [10], and the specificity and sensitivity of multiple claims with COPD diagnoses have been estimated at approximately 80% [11, 12]. The pre-existing asthma and pertussis (ASTHMA + PERT) cohort included patients with one inpatient or emergency department visit with a diagnosis of asthma or two outpatient visits with a diagnosis of asthma and at least one prescription for an asthma medication, with the first observed asthma diagnosis occurring in the 6 months before the index date. A variety of algorithms have been used to identify patients with asthma in health care claims data (e.g., [13, 14]); the algorithm used in this analysis was based on common themes of identifying patients with encounters for severe asthmatic events (inpatient or emergency department visits) or encounters for managing asthma (outpatient visits plus medication). All qualified patients identified from the database and meeting the study eligibility criteria were included in the analysis. To control for differences in underlying demographic and clinical characteristics, patients with pre-existing COPD or asthma and pertussis were matched 1:1 to patients with pertussis and no pre-existing COPD or asthma using direct covariate matching. The covariates included were patient age, sex, insurance payer, categorical Charlson Comorbidity Index (CCI) score excluding COPD (i.e., 0, 1, 2, and ⩾3), year of pertussis diagnosis, and costs in the 6 months pre-index date by quartile.

### Study measures

All-cause health care costs were calculated for three time periods: 45 days pre-index date and 45 days post-index date; 3 months pre-index date and 3 months post-index date; and 6 months pre-index date and 6 months post-index date. Pertussis-related health care costs were calculated for the post-index date time periods only. All-cause health care costs included all medical and pharmacy costs, regardless of the corresponding diagnoses or prescriptions. Pertussis-related health care costs included inpatient or emergency department visits with a primary diagnosis of pertussis; outpatient visits with a primary or secondary diagnosis of pertussis; and pharmacy claims for azithromycin, clarithromycin, erythromycin, or trimethoprim-sulfamethoxazole. Cost analyses were conducted from the total burden of disease perspective and included all payments made by the insurance provider, third parties (e.g., Medicare), and the patient. Cost data were adjusted to 2014 US dollars using the medical care component of the US Consumer Price Index [15].

### Data analyses

The incidence of diagnosed pertussis among patients with pre-existing COPD and among patients with pre-existing asthma, as well as among patients without pre-existing conditions, was calculated. Person-time (reported as person-years) accumulated during the follow-up period served as the denominator in assessing the incidence rate. The follow-up period started on the enrollment date of patients without pre-existing asthma or COPD or the exposure index date among patients with pre-existing asthma or pre-existing COPD and ended with the pertussis index date among patients with pertussis, or the end of enrollment or end of the study period (whichever came first) among patients who were not diagnosed with pertussis. Estimation of 95% confidence intervals (CIs) was based on the Poisson distribution [16, 17]. The incidence of diagnosed pertussis was calculated for patients with and without pre-existing asthma or COPD and was stratified by age. In addition, the relative risk of diagnosed pertussis among patients with each pre-existing condition compared with those without pre-existing conditions was computed and stratified by age.

Regression models were estimated to determine the adjusted excess costs during the 45-day and 3- and 6-month periods after the study index date. Adjusted health care costs were reported for each post-index date time period using a generalized estimating equation model with a log-link for the mean and a *γ* distribution for the residuals. The covariates included in the model were pre-existing COPD or asthma, sex, geographic region, health plan type, payer type, CCI score, year of diagnosis, age, and baseline costs. Missing values for certain variables (e.g., region) were treated as a separate category but were not excluded from the analysis. From the model estimations, predicted costs were generated and reported for the COPD + PERT cohort, the ASTHMA + PERT cohort, and the respective matched cohorts. Adjusted excess health care costs were reported as the adjusted costs for the COPD + PERT cohort or the ASTHMA + PERT cohort minus the adjusted costs for the respective matched cohort.

## RESULTS

### Incidence of diagnosed pertussis

Table 1 presents the incidence of diagnosed pertussis in the MarketScan database population. The overall incidence of pertussis among patients with pre-existing COPD was 0·176 (95% CI 0·166–0·185) per 1000 person-years (relative risk, 2·533 (95% CI 2·396–2·678) *vs*. patients without asthma or COPD). Among patients with pre-existing COPD, the relative risk of pertussis was highest among patients aged 19–64 years (relative risk, 3·588 (95% CI 3·353–3·840)). The overall incidence of pertussis among patients with pre-existing asthma was 0·274 (95% CI 0·265–0·285) per 1000 person-years (relative risk, 3·959 (95% CI 3·808–4·115) *vs*. patients without asthma or COPD). Among patients with pre-existing asthma, the relative risk of pertussis was also highest among patients aged 19–64 years (relative risk 4·060 (95% CI 3·861–4·269)).

**Table 1.** Incidence of diagnosed pertussis among adolescents and adults with pre-existing COPD or asthma

Cohort	*N*	Sum of follow-up time (years)	Incidence per 1000 person-years (95% CI)	Relative risk *vs*. patients with no pre-existing Asthma or COPD (95% CI)
**Overall**				
Patients with pre-existing COPD and pertussis	1313	1940	0·176 (0·166–0·185)	2·533 (2·396–2·678)
Patients with pre-existing COPD and no pertussis	2 681 930	7 476 119		
Patients with pre-existing asthma and pertussis	2917	4122	0·274 (0·265–0·285)	3·959 (3·808–4·115)
Patients with pre-existing asthma and no pertussis	3 969 136	10 625 678		
Patients with no pre-existing COPD or asthma and pertussis	20 672	48 521	0·069 (0·068–0·070)	–
Patients with no pre-existing COPD or asthma and no pertussis	124 228 622	298 178 050		
**Patients aged 11–18 years**				
Patients with pre-existing COPD and pertussis	89	75	0·423 (0·340–0·521)	3·225 (2·616–3·976)
Patients with pre-existing COPD and no pertussis	63241	210259		
Patients with pre-existing asthma and pertussis	1073	1389	0·410 (0·385–0·435)	3·122 (2·926–3·331)
Patients with pre-existing asthma and no pertussis	866 107	2 618 735		
Patients with no pre-existing COPD or asthma and pertussis	6181	14 972	0·131 (0·128–0·135)	–
Patients with no pre-existing COPD or asthma and no pertussis	20 474 781	47 100 219		
**Patients aged 19–64 years**				
Patients with pre-existing COPD and pertussis	889	1293	0·206 (0·193–0·220)	3·588 (3·353–3·840)
Patients with pre-existing COPD and no pertussis	1 515 789	4 316 406		
Patients with pre-existing asthma and pertussis	1721	2547	0·233 (0·222–0·244)	4·060 (3·861–4·269)
Patients with pre-existing asthma and no pertussis	2 863 430	7 384 314		
Patients with no pre-existing COPD or asthma and pertussis	13 200	30 392	0·057 (0·056–0·058)	–
Patients with no pre-existing COPD or asthma and no pertussis	96 930 843	230 008 831		
**Patients aged 65 + years**				
Patients with pre-existing COPD and pertussis	335	572	0·114 (0·102–0·126)	1·854 (1·644–2·090)
Patients with pre-existing COPD and no pertussis	1 102 900	2 949 454		
Patients with pre-existing asthma and pertussis	123	187	0·197 (0·164–0·236)	3·224 (2·679–3·878)
Patients with pre-existing asthma and no pertussis	239 599	622 629		
Patients with no pre-existing COPD or asthma and pertussis	1291	3158	0·061 (0·058–0·065)	–
Patients with no pre-existing COPD or asthma and no pertussis	6 822 998	21 069 001		

CI, confidence interval; COPD, chronic obstructive pulmonary disease.

### Sample characteristics

A total of 441 patients who qualified for the cohort with pre-existing COPD and pertussis (i.e., the COPD + PERT cohort) and 1157 patients who qualified for cohort with pre-existing asthma and pertussis (i.e., the ASTHMA + PERT cohort) were identified. Matching resulted in 343 patients in the COPD + PERT cohort and 1041 patients in the ASTHMA + PERT cohort. Table 2 presents the sample characteristics.

**Table 2.** Sample characteristics

	COPD + pertussis cohort (*N* = 343)		Matched cohort (*N* = 343)		Asthma + pertussis cohort (*N* = 1041)		Matched cohort (*N* = 1041)	
**Age**								
11–18 years (*n*, %)	27	7·87%	27	7·87%	453	43·52%	453	43·52%
19 to 64 years (*n*, %)	230	67·06%	230	67·06%	545	52·35%	545	52·35%
65 + years (*n*, %)	86	25·07%	86	25·07%	43	4·13%	43	4·13%
Mean (s.d.)	53·18 (18·41)		53·21 (18·40)		32·11 (19·46)		32·29 (19·35)	
Median	56·00		56·00		29·00		28·00	
Range (minimum-maximum)	11–91		11–93		11–83		11–84	
**Sex (*n*, %)**								
Male	127	37·03%	127	37·03%	379	36·41%	379	36·41%
Female	216	62·97%	216	62·97%	662	63·59%	662	63·59%
**Region (*n*, %)**								
Northeast	69	20·12%	66	19·24%	246	23·63%	245	23·54%
North Central	73	21·28%	75	21·87%	197	18·92%	242	23·25%
South	115	33·53%	91	26·53%	264	25·36%	237	22·77%
West	68	19·83%	87	25·36%	229	22·00%	215	20·65%
Missing/unknown	18	5·25%	24	7·00%	105	10·09%	102	9·80%
**Payer type (*n*, %)**								
Commercial	237	69·10%	237	69·10%	908	87·22%	908	87·22%
Medicare	91	26·53%	91	26·53%	47	4·51%	47	4·51%
Medicaid	15	4·37%	15	4·37%	86	8·26%	86	8·26%
**Most common Charlson comorbidities^a^ (*n*, %)**								
Peripheral vascular disease	26	7·58%	15	4·37%	14	1·34%	11	1·06%
Cerebrovascular disease	31	9·04%	18	5·25%	9	0·86%	23	2·21%
Diabetes without end organ damage	60	17·49%	64	18·66%	69	6·63%	71	6·82%
Depression	37	10·79%	38	11·08%	99	9·51%	94	9·03%
Hypertension	132	38·48%	112	32·65%	157	15·08%	154	14·79%
Any tumor	25	2·92%	35	10·20%	20	1·92%	21	2·02%
Skin ulcers/cellulitis	14	4·08%	28	8·16%	27	2·59%	27	2·59%
Moderate or severe liver disease	23	6·71%	28	8·16%	33	3·17%	31	2·98%
**Charlson Comorbidity Index score**								
Mean (s.d.)	1·72 (2·20)		1·8 (2·42)		0·63 (1·32)		0·65 (1·43)	
Median	1·00		1·00		0·00		0·00	
Range (minimum-maximum)	0–13		0–13		0–15		0–12	
**Year of pertussis diagnosis (*n*, %)**								
2006	15	4·37%	15	4·37%	29	2·79%	29	2·79%
2007	23	6·71%	23	6·71%	47	4·51%	47	4·51%
2008	26	7·58%	26	7·58%	93	8·93%	93	8·93%
2009	34	9·91%	34	9·91%	111	10·66%	111	10·66%
2010	57	16·62%	57	16·62%	173	16·62%	173	16·62%
2011	58	16·91%	58	16·91%	168	16·14%	168	16·14%
2012	79	23·03%	79	23·03%	272	26·13%	272	26·13%
2013	51	14·87%	51	14·87%	148	14·22%	148	14·22%
**Costs in the 6-month pre-index date period**								
Quartiles (*n*, %)								
$0-$379	6	1·75%	6	1·75%	26	2·50%	26	2·50%
$380-$1261	40	11·66%	40	11·66%	160	15·37%	160	15·37%
$1262-$3668	92	26·82%	92	26·82%	370	35·54%	370	35·54%
$3669-$ 542 638	205	59·77%	205	59·77%	485	46·59%	485	46·59%
Mean (s.d.)	$12 230 ($34 058)		$10 413 ($22 142)		$7360 ($14 464)		$6095 ($11 052)	
Median	$4805		$4392		$3119		$2958	
Range (minimum-maximum)	$72–$530 969		$78–$241 122		$0–$228 595		$0–$143 295	

COPD, chronic obstructive pulmonary disease; s.d., standard deviation.

a Charlson comorbidities measured in the 6-month pre-study index date period, and excluding COPD. The comorbidities presented affected more than 7% of any cohort or more than 2% of all cohorts. The comorbidities not presented included congestive heart failure, myocardial infarction, dementia, connective tissue disease, ulcer disease, use of warfarin, hemiplegia, moderate or severe renal disease, diabetes with end-organ damage, mild liver disease, metastatic cancer, and AIDS.

The mean (standard deviation (s.d.)) age of the COPD + PERT cohort was 53·2 (18·4) years, and approximately two-thirds of this cohort was female. The mean (s.d.) CCI score was 1·7 (2·2) for the COPD + PERT cohort *vs*. 1·8 (2·4) for the matched cohort. For both the COPD + PERT cohort and the matched cohort, the highest proportion of pertussis diagnoses occurred in 2012. Mean (s.d.) costs during the 6-month pre-index date period were slightly higher for the COPD + PERT cohort ($12 230 ($34 058)) than for the matched cohort ($10 413 ($22 142)).

The mean (s.d.) age of the ASTHMA + PERT cohort was 32·1 (19·5) years, and approximately two-thirds of this cohort was female. The mean (s.d.) CCI score was 0·6 (1·3) for patients with pre-existing asthma and pertussis *vs*. 0·7 (1·4) for matched patients. For both the ASTHMA + PERT cohort and the matched cohort, the highest proportion of pertussis diagnoses occurred in 2012. Mean (s.d.) costs during the 6-month pre-index date period were slightly higher among the ASTHMA + PERT cohort ($7360 ($14 464)) than for the matched cohort ($6095 ($11 052)).

### Hospitalizations

During the 45 days, 3 months and 6 months post-index date, the difference in the percentage of patients with at least one all-cause hospitalization was greater in the COPD + PERT cohort than in the matched cohort relative to the respective periods pre-index date (Table 3). Likewise, during all three post-index date periods, the differences in the percentage of patients with at least one all-cause hospitalization were greater in the ASTHMA + PERT cohort than in the matched cohort relative to the respective periods pre-index date.

**Table 3.** Difference in likelihood of hospitalizations after pertussis diagnosis, by time period and cohort

Time period	COPD + pertussis cohort (*N* = 343)	Matched cohort (*N* = 343)	Difference	Asthma + pertussis cohort (*N* = 1041)	Matched cohort (*N* = 1041)	Difference
**All-cause ⩾1 hospital admission (%)^a^**						
45 days	12·24	6·41	5·83	6·92	2·60	4·32
3 months	10·21	5·54	4·67	5·47	2·98	2·49
6 months	8·46	4·95	3·51	4·51	1·92	2·59
**Pertussis-related ⩾1 hospital admission (%)^b^**						
45 days	4·08	2·33	1·75	2·02	1·44	0·58
3 months	4·08	2·33	1·75	2·02	1·44	0·58
6 months	4·08	2·33	1·75	2·02	1·44	0·58

COPD, chronic obstructive pulmonary disease.

a All-cause hospitalizations reflect the difference in the percentage of patients with ⩾1 hospitalization during the 45-day, 3-month, and 6-month periods post-pertussis index date, respectively, relative to the 45-day, 3-month, and 6-month periods pre-pertussis index date.

b Pertussis-related hospitalizations reflect the percentage of patients with ⩾1 hospitalization related to pertussis in the 45 days, 3 months, and 6 months post-pertussis index date.

Similar patterns were observed for pertussis-related hospitalization patterns: across all three post-index date periods, the percentage of patients with at least one pertussis-related hospitalization were greater in the COPD + PERT and ASTHMA + PERT cohorts than in the respective matched cohorts.

### Unadjusted costs

#### Unadjusted all-cause costs

During the 45 days post-index date, the COPD + PERT cohort accrued $4150 more than they accrued during the 45 days pre-index date, whereas the matched-control cohort accrued $1201 more during the 45 days post-index date, resulting in an excess cost of pertussis in patients with COPD of $2950 (*P* = 0·009) compared with patients with pertussis without COPD (Table 4). For the 3-month and 6-month periods, the excess costs of pertussis in the COPD + PERT cohort compared with the matched-control cohort were $3499 (*P* = 0·026) and $5657 (*P* = 0·012), respectively. During the 45 days post-index date, the ASTHMA + PERT cohort accrued $1746 more than they accrued during the 45 days pre-index date, whereas the matched-control cohort accrued $1084 more during the 45 days post-index date, resulting in an excess cost of pertussis in patients with asthma compared with patients with pertussis without asthma of $662 (*P* = 0·079). For the 3- and 6-month periods, the excess costs of pertussis in the ASTHMA + PERT cohort compared with the matched-control cohort were $591 (*P* = 0·244) and $1386 (*P* = 0·129), respectively.

**Table 4.** Unadjusted mean difference in all-cause and pertussis-related costs, by time period and cohort

Time period	COPD + pertussis cohort (*N* = 343)	Matched cohort (*N* = 343)	Difference^a^ (*P* value)	Asthma + pertussis cohort (*N* = 1041)	Matched cohort (*N* = 1041)	Difference^a^ (*P* value)
**All-cause health care costs^b^**						
*45 day*s						
Overall costs	$4150	$1201	$2950 (*P* = 0·009)	$1746	$1084	$662 (*P* = 0·079)
Inpatient costs	$3675	$1147	$2529	$1060	$643	$417
Outpatient costs	$461	$93	$368	$614	$365	$249
Pharmacy costs	$14	−$39	$53	$72	$76	−$4
*3 month*s						
Overall costs	$4446	$997	$3449 (*P* = 0·026)	$1698	$1107	$591 (*P* = 0·244)
Inpatient costs	$3625	$1350	$2274	$929	$634	$295
Outpatient costs	$719	−$60	$781	$640	$449	$191
Pharmacy costs	$102	−$293	$394	$129	$24	$105
*6 months*						
Overall costs	$5858	$201	$5657 (*P* = 0·012)	$2687	$1301	$1386 (*P* = 0·129)
Inpatient costs	$4339	$1029	$3310	$1390	$962	$427
Outpatient costs	$1393	−$445	$1837	$1075	$317	$759
Pharmacy costs	$126	−$383	$510	$222	$22	$200
**Pertussis-related costs^c^**						
*45 days*						
Overall costs	$1451	$496	$956 (*P* = 0·128)	$522	$347	$175 (*P* = 0·032)
Inpatient costs	$1098	$286	$812	$178	$164	$14
Outpatient costs	$334	$195	$140	$324	$166	$158
Pharmacy costs	$19	$15	$4	$20	$17	$3
*3 months*						
Overall costs	$1475	$502	$972 (*P* = 0·121)	$541	$353	$188 (*P* = 0·021)
Inpatient costs	$1098	$286	$812	$178	$164	$14
Outpatient costs	$354	$200	$153	$339	$170	$168
Pharmacy costs	$23	$16	$7	$24	$19	$6
*6 months*						
Overall costs	$1487	$509	$979 (*P* = 0·119)	$555	$358	$197 (*P* = 0·016)
Inpatient costs	$1098	$286	$812	$178	$164	$14
Outpatient costs	$359	$204	$156	$346	$172	$174
Pharmacy costs	$30	$19	$11	$31	$22	$9

COPD, chronic obstructive pulmonary disease.

a Due to rounding, the difference may not correspond to the difference between the individual values presented for the COPD + pertussis cohort and the matched cohort.

b All-cause costs reflect the difference in costs (i.e., all medical and pharmacy costs, regardless of the corresponding diagnoses or prescriptions) incurred during the 45-day, 3-month, and 6-month periods post-pertussis index date (including pertussis-related costs), respectively, relative to the 45-day, 3-month, and 6-month periods pre-pertussis index date.

c Pertussis-related costs reflect costs associated with pertussis in the 45 days, 3 months, and 6 months post-pertussis index date.

#### Unadjusted pertussis-related costs

Compared with the matched cohort, the COPD + PERT cohort accrued $956 more in pertussis-related unadjusted costs during the 45 days post-index date (*P* = 0·128), $972 more during the 3 months post-index date (*P* = 0·121), and $979 more during the 6 months post-index date (*P* = 0·119) (Table 4). Compared with the matched cohort, the ASTHMA + PERT cohort accrued $175 more in pertussis-related unadjusted costs during the 45 days post-index date (*P* = 0·032), $188 more during the 3 months post-index date (*P* = 0·021), and $197 more during the 6 months post-index date (*P* = 0·016).

### Adjusted costs

#### Adjusted all-cause costs

**Fig. 1.**
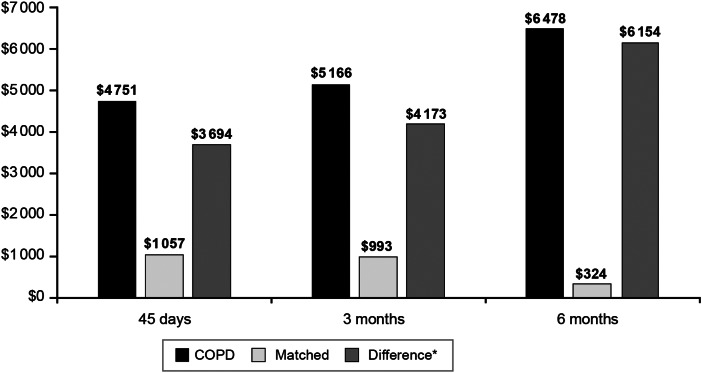
Adjusted all-cause health care costs for patients with pre-existing COPD and pertussis and matched patients with pertussis only in the 45 days, 3 months, and 6 months following the index date. COPD = chronic obstructive pulmonary disease. *P < 0·0001 for all differences.

In the 45-day period after the index date, the COPD + PERT cohort accrued $4751 in all-cause adjusted health care costs, whereas the matched cohort accrued $1057 in all-cause adjusted health care costs. This resulted in $3694 more in all-cause adjusted costs associated with the COPD + PERT cohort compared with the matched cohort (*P* < 0·0001) (Fig. 1). The difference in all-cause adjusted costs of the COPD + PERT cohort *vs*. the matched cohort increased to $4173 in the 3 months post-index date and to $6154 in the 6 months post-index date (both *P* < 0·0001). The difference in all-cause adjusted costs between the COPD + PERT cohort *vs*. the matched cohort was found to increase with age during the 45-day post-index date period ($1474 among patients aged 11–18 years, $3356 among patients aged 19–64 years, and $5295 among patients aged 65 + years; all *P* < 0·0001). The trend of increasing differences in all-cause adjusted costs with increasing patient age continued during the 3-month period ($1643 among patients aged 11–18 years, $4092 among patients aged 19–64 years, and $5184 among patients aged 65 + years; all *P* < 0·0001) and the 6-month period ($2276 among patients aged 11–18 years, $6110 among patients aged 19–64 years, and $7491 among patients aged 65 + years; all *P* < 0·0001).

**Fig. 2.**
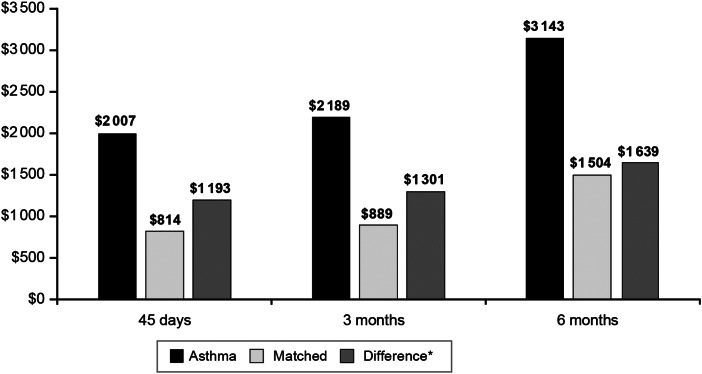
Adjusted all-cause health care costs for patients with pre-existing asthma and pertussis and matched patients with pertussis only in the 45 days, 3 months, and 6 months following the index date. *P < 0·0001 for all differences.

In the 45-day period after the index date, the ASTHMA + PERT cohort accrued $2007 in all-cause adjusted health care costs, whereas the matched cohort accrued $814 in all-cause adjusted health care costs. This resulted in $1193 more in all-cause adjusted costs associated with the ASTHMA + PERT cohort compared with the matched cohort (*P* < 0·0001) (Fig. 2). The difference in all-cause adjusted costs between the ASTHMA + PERT cohort *vs*. the matched cohort increased slightly to $1301 in the 3 months post-index date and to $1639 in the 6 months post-index date (both *P* < 0·0001). The difference in all-cause adjusted costs between the ASTHMA + PERT cohort *vs*. the matched cohort was found to increase with increasing patient age during the 45-day post-index date period ($746 among patients aged 11–18 years, $1460 among patients aged 19–64 years, and $2510 among patients aged 65 + years; all *P* < 0·0001). The trend of increasing differences in all-cause adjusted costs with increasing patient age continued during the 3-month period ($794 among patients aged 11–18 years, $1632 among patients aged 19–64 years, and $2436 among patients aged 65 + years; all *P* < 0·0001) and the 6-month period ($1021 among patients aged 11–18 years, $2087 among patients aged 19–64 years, and $2475 among patients aged 65 + years; all *P* < 0·0001).

#### Adjusted pertussis-related costs

**Fig. 3.**
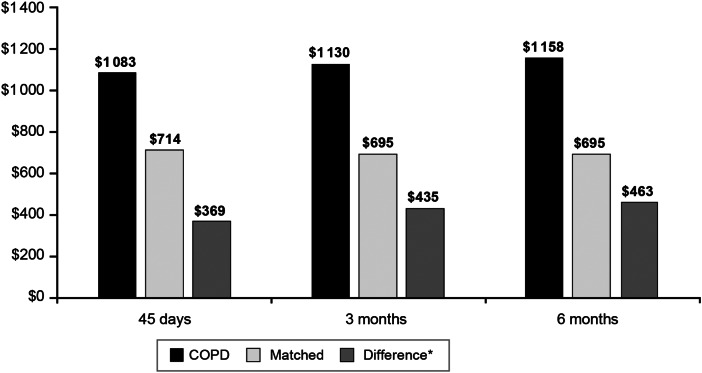
Adjusted pertussis-related health care costs for patients with pre-existing COPD and pertussis and matched patients with pertussis only in the 45 days, 3 months, and 6 months following the index date. COPD = chronic obstructive pulmonary disease. *P < 0·0001 for all differences.

The difference in pertussis-related adjusted costs between the COPD + PERT cohort and the matched cohort was $369 in the 45-day period after the index date, $435 during the 3-month period, and $463 during the 6-month period (all *P* < 0·0001) (Fig. 3). The difference in pertussis-related adjusted costs between the COPD + PERT cohort *vs.* the matched cohort during the 45-day period was found to increase with increasing patient age ($264 among patients aged 11–18 years, $361 among patients 19–64 years, and $423 among patients aged 65 + years; all *P* < 0·01). The trend of increasing differences in pertussis-related adjusted costs with age continued during the 3-month period ($281 among patients aged 11–18 years, $428 among patients aged 19–64 years, and $500 among patients aged 65 + years; all *P* < 0·01) and the 6-month period ($285 among patients aged 11–18 years, $459 among patients aged 19–64 years, and $528 among patients aged 65 + years; all *P* < 0·01).

**Fig. 4.**
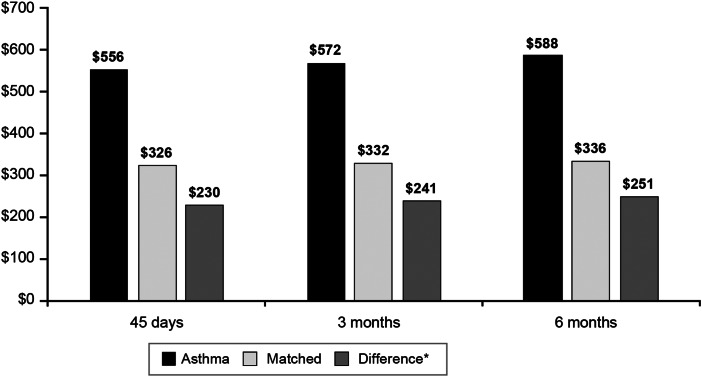
Adjusted pertussis-related health care costs for patients with pre-existing asthma and pertussis and matched patients with pertussis only in the 45 days, 3 months, and 6 months following the index date. *P < 0·0001 for all differences.

The difference in pertussis-related adjusted costs between the ASTHMA + PERT cohort and the matched cohort was $230 in the 45-day period after the index date, $241 during the 3-month period, and $251 during the 6-month period (all *P* < 0·0001) (Fig. 4). The differences in pertussis-related adjusted costs between the ASTHMA + PERT cohort *vs*. the matched cohort were similar across age groups during the 45-day period ($183 among patients aged 11–18 years, $267 among patients aged 19–64 years, and $255 among patients aged 65 + years; all *P* < 0·0001). Similar differences in pertussis-related adjusted costs between the ASTHMA + PERT cohort *vs*. the matched cohort were also observed across age groups during the 3-month period ($193 among patients aged 11–18 years, $278 among patients aged 19–64 years, and $266 among patients aged 65 + years; all *P* < 0·0001) and during the 6-month period ($204 among patients aged 11–18 years, $289 among patients aged 19–64 years, and $276 among patients aged 65 + years; all *P* < 0·0001).

## DISCUSSION

This retrospective database analysis compared patients with diagnosed pertussis and pre-existing asthma or COPD with matched patients who had diagnosed pertussis but no pre-existing asthma or COPD, and reported on the incidence of diagnosed pertussis as well as all-cause and pertussis-related costs. The results for the 2006–2014 study period may be generalizable to a large insured US population. The observed incidence rates varied considerably across age groups, with the highest incidence occurring among adolescents aged 11–18 years in both the PERT + COPD and the PERT + ASTHMA populations. Similar variations in incidence by age have been reported by the CDC during this time frame [18], but, to our knowledge, there is limited evidence on the incidence of pertussis among individuals with pre-existing asthma or COPD.

Respiratory infections such as pertussis are associated with an increase in acute exacerbations in patients with asthma and COPD [19–22]. Furthermore, some research has suggested that patients with asthma or COPD may be at increased risk for pertussis compared with patients without either of these conditions [5, 7, 8, 9]. Our study builds upon the current literature and shows that, regardless of the age group, the incidence of ICD-9-CM-diagnosed pertussis was higher among patients with a diagnosis of COPD or asthma compared with patients without either of these conditions.

Additionally, limited published data exist regarding costs associated with pertussis in adolescents and adults. A small US survey study evaluating the economic impact of pertussis among adolescents found societal costs, including direct medical and indirect costs, to be in excess of $315 per affected household [23]. Further, a study by McGuiness and colleagues [6] evaluated US patients with pertussis aged 50 years and older and reported on inpatient and outpatient health care costs using a retrospective database. Their study found that patients with pertussis accrued between $1835 and $14 428 in all-cause costs, depending on whether they received treatment in the inpatient or outpatient setting. Results from this study are not directly comparable with our analysis, which included adolescents and adults of all ages and stratified patients with pertussis by COPD and asthma status. Nevertheless, average adjusted per-patient all-cause costs in our analysis were within the range of all-cause costs identified by McGuiness and colleagues [6], ranging from $3143 for patients with asthma to $6478 for patients with COPD in the 6 months post-index date.

Our study supports previous findings that pertussis may be associated with worsening COPD and asthma symptoms, thereby increasing the overall economic burden of the diseases. Among patients with pre-existing COPD in particular, all-cause costs increased with time following a diagnosis of pertussis, whereas all-cause costs decreased over time for matched patients without COPD. These findings suggest that patients with pre-existing COPD can experience negative consequences from pertussis for at least 6 months. Specifically, matched patients without COPD accrued $1057 in adjusted costs (while controlling for costs in the pre-index date period) in the 45 days after the index date. However, adjusted costs for matched patients without COPD were found to decrease over time, with matched patients without COPD having just $324 in adjusted costs in the 6 months after the index date. This indicates that patients without COPD did not experience lingering symptoms and that the pertussis diagnosis did not have long-term health consequences for patients without pre-existing asthma or COPD. Contrasting this with patients with COPD, the burden of pertussis increased over time even after controlling for baseline health care costs, from adjusted costs of $4751 in the 45-day post-index date to $6478 in the 6-month post-index date, indicating that patients with pertussis and COPD experienced longer term negative consequences from pertussis compared to patients with pertussis without COPD. This conclusion is further supported by the fact that pertussis-related spending remained constant between the 45-day and 6-month period for patients with COPD (i.e., adjusted pertussis-related costs of $1083 in the 45-day period and $1158 in the 6-month period). Thus, patients were not accruing additional pertussis-related care after the 45-day post-index date period, and all subsequent health care expenditures causing the increase in adjusted all-cause costs over time were not directly related to the treatment of pertussis but rather were associated with increased health care costs due to other concomitant conditions. Similar findings were also observed for asthma patients with pertussis; however, the economic consequences were not as large (range: adjusted all-cause costs of $2007 in the 45-day post-study index period to adjusted all-cause costs of $3143 in the 6-month post-study index period).

Tetanus, diphtheria, and acellular pertussis (Tdap) vaccination remains the most effective strategy against infection and in reducing disease severity among adolescents and adults. Protective benefits of Tdap vaccination have ranged from an estimated effectiveness of 53·0% among patients aged 11 years or older in the community setting [24] to an estimated efficacy of 92% among adults participating in the Adult Pertussis Trial [25]; however, recent research has found that this protection from pertussis wanes over time [26, 27]. Although there are no studies specifically examining Tdap vaccine efficacy among patients with COPD or asthma, whole-cell pertussis vaccination has been shown to have a protective effect against allergic asthma exacerbations [28]. In addition, other vaccines, such as the influenza vaccine, are associated with a reduction in acute exacerbations among patients with COPD [29]. Therefore, incorporating compliance with the Advisory Committee on Immunization Practice (ACIP) Tdap recommendations into COPD and asthma management plans may aid in reducing morbidity and mortality among these patients. The ACIP currently recommends one dose of Tdap vaccine for non-pregnant adolescents (preferred at ages 11 or 12 years) and adults [30]. However, compliance in the general adult population is low; in 2014, an estimated 20·1% of adults had received the Tdap vaccine in the previous 9 years [31]. Given the lack of information on the effectiveness of Tdap in patients with COPD and asthma, further research on the measurable effect of vaccination on infection and disease severity is warranted.

This study has several limitations common to retrospective database analyses, including the potential for coding errors in the administrative claims data and for selection bias. In particular, some of the 27 adolescent patients (aged 11–18 years) in the study sample who had COPD may have been subject to diagnostic coding errors. Although observable measures were used to alleviate the effect of selection bias by conducting direct covariate matching, unmeasured or unknown factors may have affected the outcome differently. Moreover, data on laboratory-confirmed pertussis are unavailable in the MarketScan databases; therefore, pertussis cases were identified using diagnosis codes, which likely resulted in an underestimation of the number of patients with pertussis, especially in adults. Similarly, spirometry testing results were not available, and identification of COPD and asthma also relied on established claims-based algorithms. Additionally, this analysis may have underestimated the incidence and burden of pertussis, as some patients may not have sought care with a health care provider. Finally, some non-pertussis-related illnesses were included when calculating all-cause costs (although because the analysis evaluated the change in costs between the pre- and post-index date periods, any non-pertussis-related costs that occurred in the post-index period would reasonably have been expected to be balanced out by those in the pre-index date period).

To conclude, this study identified the potential for long-term complications from pertussis among patients with pre-existing respiratory conditions. Additional explorations of disease course and specific outcomes following pertussis infection, including complications and resource-use patterns, in individuals with respiratory comorbidities are warranted. During the 6-month follow-up window after a diagnosis of pertussis, patients with pertussis and COPD accrued approximately $6154 more in adjusted health care costs compared with matched patients with pertussis without COPD or asthma, while patients with pertussis and asthma accrued approximately $1639 more in adjusted health care costs compared with matched patients with pertussis without COPD or asthma. Additionally, this study found that patients with pre-existing asthma or COPD were more likely to have diagnosed pertussis compared with patients without pre-existing asthma or COPD. Focused interventions to prevent pertussis infection among these high-risk patients, including targeted Tdap vaccination strategies, could aid in reducing exacerbation of asthma and COPD, resulting in long-term health care savings.
